# Enriched CO_2_ and Root-Associated Fungi (Mycorrhizae) Yield Inverse Effects on Plant Mass and Root Morphology in Six *Asclepias* Species

**DOI:** 10.3390/plants10112474

**Published:** 2021-11-16

**Authors:** Rondy J. Malik, James D. Bever

**Affiliations:** Department of Ecology and Evolutionary Biology, Kansas Biological Survey, 2101 Constant Ave, Lawrence, KS 66045, USA; jbever@ku.edu

**Keywords:** CO_2_, *Asclepias*, mycorrhizae, root morphology

## Abstract

While milkweeds (*Asclepias* spp.) are important for sustaining biodiversity in marginal ecosystems, CO_2_ flux may afflict *Asclepias* species and cause detriment to native communities. Negative CO_2_-induced effects may be mitigated through mycorrhizal associations. In this study, we sought to determine how mycorrhizae interacts with CO_2_ to influence *Asclepias* biomass and root morphology. A broad range of *Asclepias* species (n = 6) were chosen for this study, including four tap-root species (*A. sullivantii, A. syriaca*, *A. tuberosa*, and *A. viridis*) and two fibrous root species (*A. incarnata* and *A. verticillata*). Collectively, the six *Asclepias* species were manipulated under a 2 × 2 full-factorial design that featured two mycorrhizal levels (−/+ mycorrhizae) and two CO_2_ levels (ambient and enriched (i.e., 3.5× ambient)). After a duration of 10 months, *Asclepias* responses were assessed as whole dry weight (i.e., biomass) and relative transportive root. Relative transportive root is the percent difference in the diameter of highest order root (transportive root) versus that of first-order absorptive roots. Results revealed an asymmetrical response, as mycorrhizae increased *Asclepias* biomass by ~12-fold, while enriched CO_2_ decreased biomass by about 25%. CO_2_ did not impact relative transportive roots, but mycorrhizae increased root organ’s response by more than 20%. Interactions with CO_2_ and mycorrhizae were observed for both biomass and root morphology (i.e., relative transportive root). A gene associated with CO_2_ fixation (*rbcL*) revealed that the two fibrous root species formed a phylogenetic clade that was distant from the four tap-root species. The effect of mycorrhizae was most profound in tap-root systems, as mycorrhizae modified the highest order root into tuber-like structures. A strong positive correlation was observed with biomass and relative transportive root. This study elucidates the interplay with roots, mycorrhizae, and CO_2_, while providing a potential pathway for mycorrhizae to ameliorate CO_2_ induced effects.

## 1. Introduction

Plants have a broad range of traits that vary in response to environmental cues [1,2], including atmospheric carbon. CO_2_-induced responses (i.e., guard cell regulation and stomatal conductance) have long been characterized [3,4], while subsurface responses, particularly those pertaining to roots, have become increasingly controversial [5,6,7,8]. Roots are conduits for carbon flux, as these extensive organs can modulate soil chemistry, structure, and microbes [9,10,11,12]. At the root–soil interface, mycorrhizal associations are sustained through carbon–nutrient exchange [13,14], but a rise in atmospheric carbon may strain root-mediated traits [6,15], and eco-physiological function (i.e., root respiration, longevity, and turnover), which are central to root-mediated C cycling [16,17,18]. CO_2_ enrichment may increase root hierarchal branching, specific length, stele, cortex, and diameter [19,20], but it is unclear how root morphology varies in response to CO_2_, especially across root order [17] and system (tap- versus fibrous root system). Roots that correspond to the hyper-fibrous end of the tap- versus fibrous root trait continuum, are less mycorrhizal dependent [21], which can lead to negative interactions (i.e., competition) with mycorrhizal extramatrical hyphae [22]. Unraveling how mycorrhizae and atmospheric carbon may interact with roots can potentially provide insight into CO_2_-induced changes.

Root morphology and CO_2_ may interact with mycorrhizae. Upon C fixation in the shoot, carbon is translocated to roots and metabolized by mycorrhizae [23], but among *glomeromycotans*, CO_2_ enrichment may not necessarily lead to an increase in arbuscule development [24]. Arbuscules are confined to root cortical cells, and are important for carbon–nutrient exchange in colonized roots. Tap- and fibrous root systems are architecturally different, but have unique advantages. Fibrous roots are conducive toward water infiltration and reducing soil erosion [11,25], while tap-root systems excel at penetrating compacted soil layers [26]. In response to CO_2_ flux, fibrous and tap-root systems may vary in modes of nutrient foraging that are independent of mycorrhizal networks. Fibrous root systems are premier foragers at soil depths proximal to organic layers [27]. Meanwhile, tap-root systems are effective at exploiting deeper soil horizons that have accumulated leached nutrients (i.e., NO_3_^−^, SO_4_^2−^), minerals [27,28], and ground water [29]. In comparison, fibrous root systems have high absorptive capacity [30,31] and specific root length (SRL) [32].

Root function is a product of root branching order [33], but still, root morphology may be impacted by CO_2_. An increase in atmospheric CO_2_ has been reported to improve lateral and tap-root sizes [34]. Tap-roots are of a higher root order in contrast to absorptive roots (i.e., root order 1 and 2), which are of a lower branching order [35]. Lower order roots are more involved in nutrient uptake and mycorrhizal associations. In contrast, higher order roots (i.e., root order > 3) have higher C–N [36], which may suggest a greater response to enriched CO_2_.

To date, very few studies have addressed the combinatorial effect of CO_2_ and mycorrhizae on roots, and even fewer studies have addressed these factors in the context of tap- versus fibrous root systems. Here, we address the following questions, (1) are the combinatorial effects of CO_2_ and mycorrhizae additive or interactive? (2) Does the outcome of CO_2_ and mycorrhizae differ in tap- versus fibrous root systems? Here, it is hypothesized that (a) the effect of CO_2_ and mycorrhizae will not be additive, as roots are likely to trade-away excess carbon to mycorrhizae. It is also hypothesized that, (b) the root response to mycorrhizae and CO_2_ will differ across the tap- versus fibrous root trait continuum, particularly due to difference in root architecture.

## 2. Material and Methods

### 2.1. Study System

The aim of this study was to determine ways in which CO_2_ and mycorrhizae can influence root morphology and plant performance (i.e., whole biomass). Thus, 6 species of milkweed from genus *Asclepias* were chosen for this experiment (Table 1). Milkweeds are excellent specimens for environmental manipulation (i.e., growth chamber) because they are adapted to a wide range of ecological niches [37], including disturbed, pristine, drought, and wetland habitats (Table 1). In addition, milkweed (hereafter referred to as *Asclepias* species) are important to marginal ecosystems (i.e., remnant prairie), biodiversity (i.e., monarch butterflies), and conservation interests.

### 2.2. Source of Asclepias spp. (Milkweed) and Mycorrhizae

*Asclepias* seeds were sourced from the midwestern region of the U.S.A. The 6 *Asclepias* spp. were *A. sullivantii, A. verticillata* (seed source, Prairie Moon Nursery Winona, MN, U.S.A), *A. incarnata*, *A. syriaca*, *A. tuberosa*, and *A. viridis* (seed source, Missouri Wild Flower Nursery (Jefferson City, MO, U.S.A). The relatedness of each of these species was characterized using a chloroplast DNA sequence that codes for ribulose bisphosphate carboxylase (*rbcL*), a conserved gene involved in CO_2_ fixation. Since *Asclepias* spp. response to (CO_2_) manipulation is the nature of this study, assessing *Asclepias* relatedness with respect to *rbcL* gene was appropriate.

Similar to *Asclepias* spp., mycorrhizal isolates were also sourced from the midwestern region of the U.S.A. Fungal consortia featured 4 isolates of mycorrhizae derived from Kankakee sands prairie reserve in Indiana, USA. Isolates included several species (*Claroideoglomus* *claroideum*, *Racocetra* *fulgida*, *Funneliformis* *mosseae*, and *Claroideoglomus* *lamellosum*) that have comparable levels of colonization [42]. In addition, these species are representative of mycorrhizal phylogenetic diversity [43,44], and include isolates of high and low experimental usage rate [45].

### 2.3. Preparing Mycorrhizal Inoculate

Mycorrhizae are lab isolates that were first isolated from prairie soil to start lab cultures (Table S1). Identity of spores were previously determined using morphological and phylogenetic species concept [43]. Since then, these isolates have been of standard use in the lab [42,46,47]. Prior to the experiment, fungal cultures were prepared and bulked on sorghum roots for a full growing season under glasshouse conditions [46]. Briefly, after a full growing season, aboveground sorghum tissue was removed, while the belowground soil and mycorrhizal root mix was stored at 4 °C prior to experimental use.

At the time of experimental use, mycorrhizal fungal inocula (fungal spores and sorghum root mix) was used to inoculate in between the top and bottom layers of heat sterile background soil (Kansas clay loam–sand (1:1)). The background soil was mixed with sand, primarily because sand enhances drainage in pots, reduces nutrient levels, and enhances plant’s mycorrhizal functional response. The volume of the added inocula (i.e., fungal spore–root mix) was 50 cm^3^ (+ mycorrhizae). Meanwhile, sterile inocula void of live mycorrhizae was added to control pots (− mycorrhizae), also at a volume of 50 cm^3^. This assured that the soil structure and nutrient ratio of all pots was maintained. Finally, *Asclepias* seedlings were transplanted to cone-tainer pots (Stuewe & Sons Inc., Tangent, Oregon, U.S.A.), which were positive or negative for mycorrhizae.

### 2.4. Growth Chamber and Atmospheric Conditions

Prior to transplanting milkweed to experimental conditions (i.e., −/+ mycorrhizae and CO_2_ manipulation), *Asclepias* spp. were germinated on heat sterile potting soil (Berger bark mix BM 7, www.berger.ca. access date: 9 September 2019) After five weeks, 5-week-old seedlings were transferred to pots that were positive or negative for mycorrhizae. The dimensions of the pots were 6.8 cm in diameter by a depth of 35.56 cm. In a random block design, pots were then placed into 4 Conviron CMP 6050 growth chambers (Controlled Environments Limited, Winnipeg, Manitoba, Canada). Two of the chambers were set to ambient (CO_2_) at 400 ppm, while the other two chambers were set to 3.75× ambient levels. The 3.75× ambient level (hereafter referred to as enriched CO_2_) was chosen because geological time records suggest (CO_2_) > 1500 ppm reduces plant stomatal index [48], which is of detriment to photosynthesis and Earth’s biosphere. Growth chamber settings were 23 °C, 70% humidity (% RH), and a lighting intensity of 1000 unit micromoles. CO_2_ concentrations were set to 400 ppm (ambient) and 1500 ppm (enriched). Plants were fertilized with nitrogen (0.2 g/L) periodically (average of once a month) at an application volume of 50 mL. Similar to Malik et al. (2016) [46], this (N) was determined and applied by using the atomic mass of N in the NH_4_NO_3_ compound, which was weighted to control for N mass. Using this ratio, we determined how much NH_4_NO_3_ is needed in 1 L of water to create 0.2 g/L of N.

### 2.5. Experimental Design

Experimental design featured 6 *Asclepias* spp. × 2 mycorrhizal levels (+/− mycorrhizae) × 2 CO_2_ conditions (ambient and 3.75× ambient) × 8 replicates per experimental treatment. In totality, the experiment was 192 observations (i.e., 192 cone-tainers). The experiment was performed under growth chamber conditions in a random block design. For 10 months, 6 *Asclepias* spp. were grown on −/+ mycorrhizal soil regimes, while under ambient and enriched (3.75× ambient) CO_2_ conditions. Plants were first germinated on heat sterile potting soil. After 5 weeks, seedlings were transplanted to −/+ mycorrhizal microcosms. The *Asclepias*–mycorrhizal microcosms were exposed to either ambient or enriched (CO_2_) for a duration of 10 months (December 2019–October 2020).

### 2.6. Harvest

After a duration of 10 months, the effects of mycorrhizae and CO_2_ on *Asclepias* spp. were observed. Roots were washed and scanned for root morphological responses. Briefly, roots were blot dried and positioned next to a scale, and an EPSON scan was used to make morphological observations. Roots were then stored in 75% ethanol and boiled in KOH, prior to trypan blue staining [49]. Staining and qualitative microscopic examination confirmed the mycorrhizal treatments. Finally, plant performance was assessed as whole dry weight.

### 2.7. Root Image Analysis

For image analysis, roots were washed and scanned to assess morphological response to CO_2_ and mycorrhizae. Root morphological response was quantified with *ImageJ* *1.46r* [50], as pixels were calibrated to centimeters to assess diameter. Root diameter was scored at the midpoint of the highest order root (transport root), as well as the midpoint of the lowest order root (absorptive root). Percent difference in transport versus absorptive root diameter (relative transport root) was estimated.

Relative transport root, or percent difference between transport and absorptive root, was appropriate because root diameter has been shown to vary across plant groups [1], including tap- and fibrous root species. Relative transport root is calculated according to Equation 1, where transport (*T*) and absorptive (*A*) root diameters are divided by transport root (*T*) diameter. This value is then multiplied by 100.

$$Relative\;transport\;root=\left(\frac{T-A}{T}\;\right)*\;100$$

### 2.8. Statistical Analysis for Root Morphology and Biomass

This study was analyzed in *R version 4.0.2.* Shapiro–Wilk and Levene’s Test, as well as diagnostic plots, were used to assess homogeneity in variance and normality for dependent variables (i.e., relative transport root and biomass). CO_2_ level, mycorrhizae, species and growth chamber effects were set as predictors, while both relative transportive root and biomass were set as response variables. MANOVA analysis was employed, as relative transport root and biomass were response variables from the same set of plants. Briefly, for MANOVA analysis, relative transport root and biomass were made into a vector, and simultaneous inferences were made by the predictor variables. The analysis was followed by a set of ANOVAs that independently predict biomass and root morphology. Interactions among the predictors were also included in the model, this was critical for determining whether or not growth chamber(s) were a confounding factor. Post-hoc analysis included Step AICc, Tukey HSD, and a priori contrast using the ‘multcomp’ package. The ‘multcomp’ package allowed simultaneous test for general linear hypotheses [51]. Finally, a correlation between relative transport root and biomass was assessed using the ‘rcorr’ and ‘cor.test’ function via ‘Hmisc’ package [52]. Correlations were then examined with 95% CI ellipses, using ‘ggplot 2’. The ellipses enabled graphical analysis for outcome and experimental variables

### 2.9. Phylogenetic Analysis: Asclepias Relatedness Using CO_2_ Fixation Gene

Amino acid (AA) sequence for *rbcL* locus was aligned using Muscle, which assesses distance using kmer distance for unaligned pairs and kimura distance for aligned pairs (Edgar, 2004). Evolutionary analysis was inferred using Maximum Likelihood Method and the JTT matrix-based model [53]. The phylogenetic tree for the 6 *Asclepias* species was constructed according to the highest log likelihood. This enabled a tree that was drawn to scale with branch lengths measured as the number of substitutions per site. While this analysis involved 6 AA sequences, there were a total of 475 positions in the final dataset. This allowed evolutionary analysis to be conducted with MEGA X [54].

## 3. Results

### 3.1. Multivariate Analysis, Qualitative Observations, and Root System

**Figure 1:**
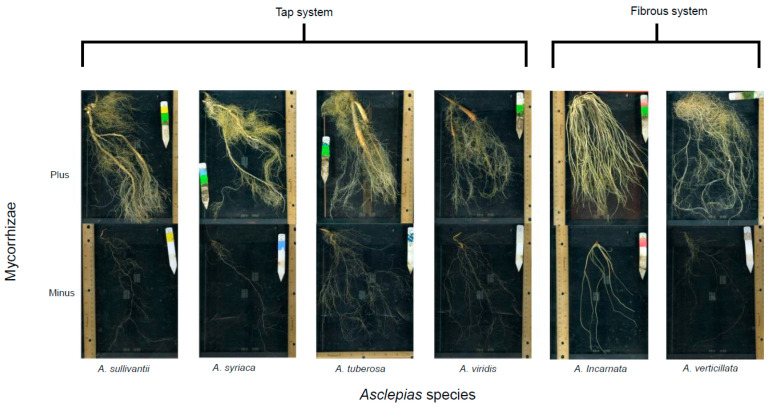
Imagery shows a subset of plants from the experiment. The top panel represents roots with mycorrhizae, the bottom panel represent roots void of mycorrhizae. Mycorrhizae had pronounced effects on root systems. In tap-root systems, the dominant root was more likely to be modified into a tuber-like structure.

The viability and effectiveness of the inoculum was confirmed after 10 months, as root–mycorrhizal structures (i.e., arbuscules and vesicles) were observed to be present in experimental roots (i.e., + Mycorrhizae), while the presence of these structures was undetected in the control. MANOVA analysis revealed that the combination of root diameter and biomass significantly responded to CO_2_ (*Pillai* _1143_ = 0.057, *p* = 0.01) and mycorrhizae (*Pillai*_1143_ = 0.064, *p* < 0.0001). Additionally, the combination of these traits led to significant interactions with CO_2_ and mycorrhizae (MANOVA, *Pillai* _1143_ = 0.051, *p* = 0.023), CO_2_ and *Asclepias* species (MANOVA, *Pillai* _5143_ = 0.140, *p* = 0.020), and mycorrhizae and *Asclepias* species (MANOVA, *Pillai* _5143_ = 0.613, *p* < 0.0001). Both fibrous root species and tap-root species were enhanced by mycorrhizae (Figure 1). In tap-root systems, dominant pronounced roots (i.e., higher order transport roots) were likely to be modified into tuber-like structures while in the presence of mycorrhizae (Figure 1). *A priori* contrast revealed significant biomass difference in species with tap- versus fibrous root systems. The difference was about 7 g (95% CI _[−∞, −2.576]_, *p* = 0.005, ANOVA, simultaneous tests for GLH, fibrous versus tap-root system). Similarly, root systems differed in relative transport roots by an estimate of 25.7% (95% CI _[−∞, −3.459]_, *p* = 0.028, ANOVA, simultaneous tests for GLH, fibrous versus tap-root system).

### 3.2. Biomass Response to Mycorrhizae and CO_2_

**Figure 2:**
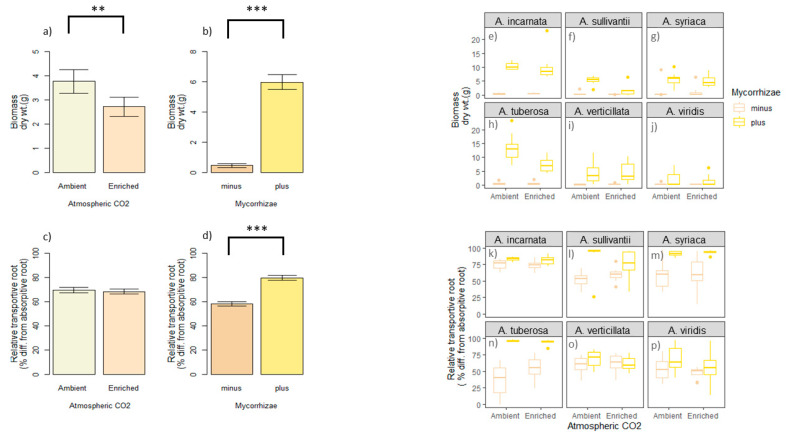
The outcome of mycorrhizae and CO_2_ (predictors) on explanatory variables (i.e., relative transportive root and biomass). Relative transportive root response is the percent difference in transportive root versus absorptive root diameter. Biomass response is dry weight in grams. The main effect of predictors on biomass are shown in (**a**,**b**). The main effect of the predictors on root response are shown in (**c**,**d**). *Asclepias* spp. biomass response to CO_2_ and mycorrhizae (i.e., predictors) are shown in (**e**–**j**)). *Asclepias* spp. root response to CO_2_ and mycorrhizae (i.e., predictors) are shown in (**k**–**p**). Error bars are standard error in (**a**–**d**). Significant codes: ‘***’ *p* < 0; ‘**’ *p* < 0.001.

Biomass (i.e., dry weight) was decreased under enriched atmospheric CO_2_ by ~25% (Figure 2a, ANOVA, *F*_1143_ = 8.525, *p* = 0.004). In contrast, mycorrhizae significantly improved biomass by ~12-fold (Figure 2b, ANOVA *F*_1143_ = 235.161, *p* < 0.0001,). Increase in biomass was broadly observed across the six *Asclepias* species (Figure 2e–j). Interestingly, biomass response was also influenced by CO_2_ and mycorrhizae interaction (ANOVA *F*_1143_ = 5.690, *p* = 0.01, Table S2), as well as CO_2_ and *Asclepias* species interaction (ANOVA, *F*_5143_ = 8.021, *p* = 0.03, Table S2).

### 3.3. Transport Root Response to Mycorrhizae and CO_2_

With regard to the relative transport root, CO_2_ did not play a significant role (Figure 2c, ANOVA, *F* _1143_ = 0.228, *p* = 0.63, Table S3), while mycorrhizae did (Figure 2d, *F*_1143_ = 91.881, *p* < 0.0001, Table S3). Specifically, mycorrhizae improved relative transport root diameter by about 20% (Figure 2d). The relative transport root was also significantly impacted by a CO_2_ and mycorrhizae interaction (*F* _1144_ = 4.580, *p* = 0.034, Table S3), as well as mycorrhizae and *Asclepias* species interaction (*F* _1143_ = 9.527, *p* < 0.0001, Table S3). In general, the relative transport root differed across *Asclepias* spp. (Figure 2k–p, *F* _5143_ = 8.937, *p* < 0.0001).

### 3.4. Correlation with Relative Transport and Biomass

**Figure 3:**
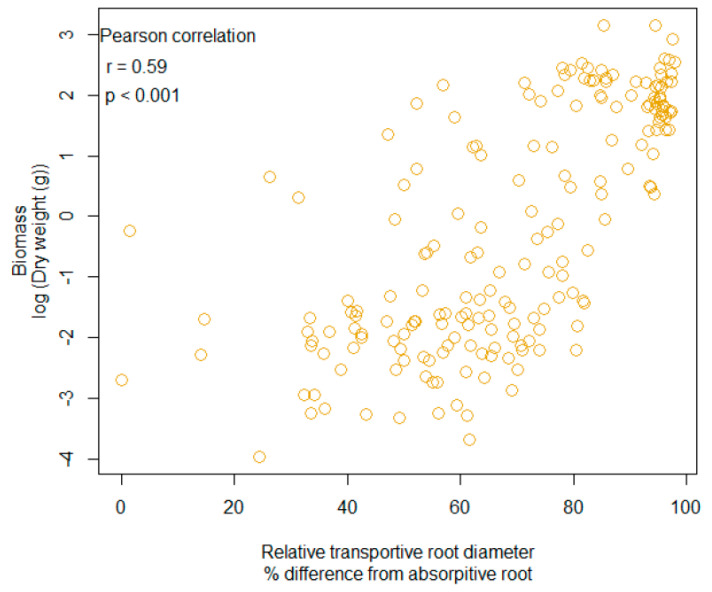
The relationship with biomass and relative root diameter. Each point represents an individual plant from the experiment.

**Figure 4:**
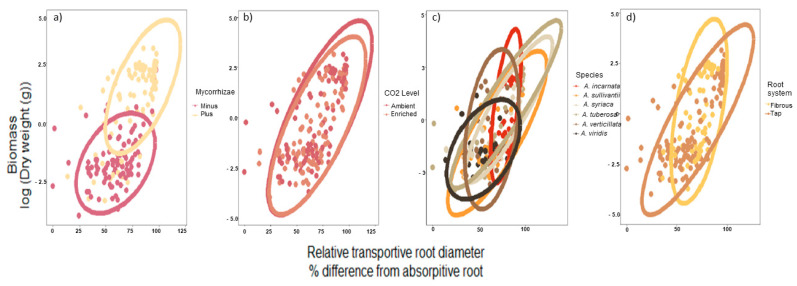
The relationship with biomass and relative transportive root across experimental factors. This relationship is decoupled by factor (**a**) mycorrhizae; (**b**) CO_2_ regime; (**c**) species; (**d**) root systems. Ellipses represent 95% CI for the levels in each of the factors described.

A significant correlation was also detected with biomass and root diameter, as these two responses were positively associated (Pearson correlation, r = 0.59, n = 191, *p* < 0.001, Figure 3). In addition, 95% CI ellipse revealed that the association with relative transport root and biomass was increasingly positive in the presence of mycorrhizae, which led to a small overlap between + mycorrhizae and − mycorrhizae (Figure 4a). The CO_2_ regime (i.e., ambient versus enriched) produced a 95% CI ellipse with a high degree of overlap and similar shape. However, the CO_2_ ellipses were not congruent as the ambient CO_2_ ellipse was more positive (Figure 4b). As it relates to species, both fibrous root species, *A*. *incarnata* and *A. verticillata* produced *ellipses* shapes that were unique and not angled at 45 degrees, unlike the tap-root species (Figure 4c). These differences are even more apparent when ellipses are assorted by root system (Figure 4d).

### 3.5. Asclepias Species Relatedness and Root System

**Figure 5:**
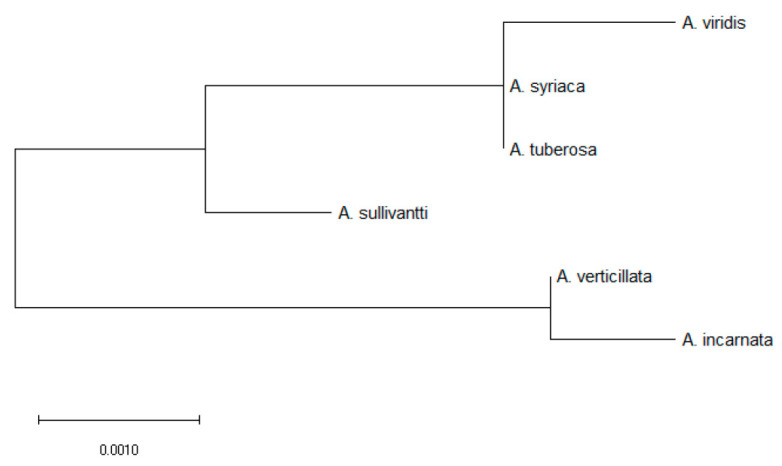
Provided here is a maximum likelihood tree for 6 the *Asclepias* species. This tree was constructed using highest log likelihood (−1398.00) for the *rbcL* amino acid sequence (*rbcL* is a gene involved in CO_2_ fixation). According to this tree, fibrous root species, *A. verticillata* and *A. incarnata*, are distant from the 4 tap-root species.

With respect to the *rbcL* locus (i.e., gene involved in CO_2_ fixation), a tree was constructed to the highest log likelihood (−1398.00) to examine relatedness of the six *Asclepias* species. Interestingly, the root system helped explain relatedness. Specifically, the two fibrous root species, *A. incarnata* and *A. verticillata* were sister taxa, and an outgroup, that was distant to the four other tap-root species (Figure 5).

### 3.6. Chamber Effects

Predictors (i.e., CO_2_, mycorrhizae, *Asclepias* species) did not interact with CO_2_ chambers (Tables S2 and S3, *p* > 0.05). This provides confidence that results were not confounded by the experimental apparatus. In addition, Step AICc revealed that response variables including biomass and relative transport root were best explained when growth chamber interactions were removed from the model.

## 4. Discussion

Enriched CO_2_ and mycorrhizae may be additive or opposing forces helping shape plant eco-physiological responses. CO_2_ flux is relevant to root life span, production, and diameter [55,56,57], as well as ecosystem processes [58,59,60], and plant fitness [61]. Interestingly, the targeted role of roots may be enhanced by mycorrhizae [62], but our findings reveal that enriched CO_2_ reduced plant biomass by 25% (Figure 2a). We speculate that the mechanism at play may be CO_2_ regulation of stomatal density, conductance, and aperture [63], as enriched CO_2_ can lead to stomatal closures, which can reduce carbon capture and net CO_2_ assimilation [64,65]. Together, this may explain negative CO_2_-induced effects on *Asclepias* spp., particularly biomass. Contrarily, mycorrhizae improved biomass by 12-fold while modifying root morphology (Figure 1 and Figure 2b,d). Significant interactions with CO_2_ and mycorrhizae were detected for biomass and relative transport roots (Tables S2 and S3). This study elucidates potential mechanisms in which CO_2_ and mycorrhizae may yield asymmetrical outcomes on plant eco-physiology.

### 4.1. Biomass, CO_2_ and Mycorrhizae

Carbon is captured from the atmosphere, and used to build biomass and organs (i.e., roots), but this would not be sustainable without root absorption, subsurface foraging, nutrient transport, and organic content storage [66,67,68]. While root functionality can be stimulated by CO_2_ [69], enriched CO_2_ can have negative outcomes on fine lateral roots and plant mass in agroecosystems [70,71]. As it relates to *Asclepias* species, which are essential to specialist herbivores (e.g., monarch butterflies), biodiversity, and prairie ecosystems; enriched CO_2_ decreased plant biomass by 25% (Figure 2a). These differences were most apparent in five of the six *Asclepias* species (*A. incarnata*, *A* *tuberosa*, *A. sullivantii*, *A. viridis*, *A. syriaca*, Figure 2e–j (but not Figure 2i)). Fibrous root species, *A. verticillata*, was the only species in which the combination of mycorrhizae and enriched CO_2_ was observed to have an additive effect on biomass (Figure 2i). This may be explained by the fact that *A. verticillata* is considered to have some of the most divergent traits in genus *Asclepias* [39]. Hence, the disparate thin pointy leaves and very fine shallow grass-like roots makes *A. verticillata* distinct, and may have a role in explaining *A. verticillata’s* unique response to mycorrhizae and CO_2_.

Interestingly, the CO_2_ effect on biomass and root morphology may be context dependent. At a CO_2_ level of 650 ppm, plant growth and percent mycorrhizal colonization was reportedly increased [72]. Perhaps suggesting that when CO_2_ stimulates plant growth, percent mycorrhizal colonization is also stimulated. While percent mycorrhizal colonization was not quantified in this study, it may be the case that since enriched CO_2_ depressed plant growth (Figure 2a), percent mycorrhizal colonization was also depressed. This may help explain the significant interaction with CO_2_ and mycorrhizae on biomass (Table S2). However, the outcome of CO_2_ on plant–mycorrhizal nutrient exchange may be species or cultivar specific [24].

### 4.2. CO_2_–Mycorrhizae Interaction and Root Response

Although elevated CO_2_ can lead to roots of high tissue density [73], root morphology has also been found to be associated with aboveground traits [1]. However, the precise mechanism as to how aboveground environmental cues affect belowground physiology remains obscure. In the present study, CO_2_ did not influence root morphology (Figure 2c), as these findings differed from a recent study [69]. It may be the case that CO_2_ can indirectly affect root morphology by increasing mycorrhizal root length colonization [74]. Interestingly, this hypothesis would rely on root systems that are highly responsive to mycorrhizae, as well as atmospheric–edaphic environment interactions.

Irrespective of the main effect of CO_2_, we observed an interaction with CO_2_ and mycorrhizae on root response (*p* = 0.03, Table S3). This may provide insight as to why the effect of plus mycorrhizal treatment depended on the CO_2_ level in *A. verticillata* roots. Specifically, mycorrhizae and enriched CO_2_ depressed *A. verticillata’s* relative transport root (Figure 2o). This sort of interaction was not observed in *A. incarnata*, the other fibrous root species. Differences among these two fibrous root species (i.e., *A. verticillata* and *A. incarnata*) may be due to divergent life histories and localized adaptation. In particular, *A*. *verticillata* thrives in xeric soils, while *A. incarnata* thrives in hydric soils (Table 1). Despite this, *A.* *incarnata* and *A. verticillata* (two fibrous root species), were more closely related than the other four tap-root species.

Tap- versus fibrous root systems may have been a good predictor of *Asclepias* spp. relatedness, as this observation does not appear to be confounded by rhizome trait(s). Hence, when rhizome traits were mapped onto a pre-existing phylogeny [75,76], it was determined that *Asclepias* spp. relatedness did not assort according to rhizome morphology. However, as it relates to the present study, *Asclepias* spp. assort according to the root system (Figure 5), as relatedness was characterized using the *rbcL* locus (Table S4)

### 4.3. Mycorrhizae, Root Modification, and Carbon Storage

Root morphology can vary across plant groups (i.e., lilies, forbes, grasses) of the same community [1]. This may be explained by root metabolomics [77], which have been shown to be altered by mycorrhizae [78]. Despite this, mycorrhizal implications may depend on the root system and morphology [21]. The outcome of mycorrhizae on root morphology was most exaggerated in tap-root systems (Figure S1), irrespective of [CO_2_]. In most cases, tap-roots were modified into tuber-like structures (Figure 1), perhaps re-iterating the notion that tap-root systems are more likely to be mycorrhizal responsive. In contrast, fibrous root systems may antagonize mycorrhizae, as fibrous root systems have been shown to have negative effects on mycorrhizal hyphal length [22]. In addition, mycorrhizal dependence has been reported to decrease with the increase in root fibrousness [21].

As it relates to the ecosystem function, mycorrhizae may use different mechanisms to promote carbon storage depending on the plant root system. Our findings suggest that in tap-root systems, mycorrhizae may directly promote root carbon storage by helping to modify tap-roots into tuber-like structures (Figure 1). However, mycorrhizae may complement fine roots by effectively promoting microaggregate stabilization [22], which can affect C sequestration and nutrient cycling [79,80,81].

## 5. Conclusions

Mycorrhizae enhanced relative transportive root and biomass, while enriched CO_2_ had the opposite effect. Interactions with CO_2_ and mycorrhizae were observed for both biomass and relative transport root. The findings of this study shed insight on how CO_2_ and mycorrhizae may interact and influence plant eco-physiology. In particular excess CO_2_ (i.e., 1500 ppm) can depress plant productivity, while mycorrhizae can act as a countering force to improve productivity.

## Figures and Tables

**Table 1:** Root system, milkweed, and qualitative observations of the 6 *Asclepias* species.

Root System	Species	Common Name	Niche	Experimental Observation
*Fibrous*	*A*. *incarnata*	Swamp milkweed	Prefers moist wet soils (wildflower.org), and is found in standing water several months of the year [38]. In addition, many stems arise from a single root stock [38].	Fine roots became increasingly coarse when colonized with mycorrhizae.
	*A*. *verticillata*	Whorled milkweed	Has divergent traits including shallow fine roots that are 5–10 cm deep [39]. Additionally, prefers xeric soils (Missouri botanical garden.org), and grows extensive rhizome systems [38].	Very dense fine roots that tightly aggregated the soil together when inoculated with mycorrhizae.
*Tap*	*A*. *syriaca*	Common milkweed	Prefers well-drained loamy soils (wildflower.org). Grows large extensive rhizome systems while adjacent stems may belong to the same or different clones [38].	Primary/dominant root is modified into tuber-like structures when colonized with mycorrhizae.
	*A*. *sullivantii*	Prairie milkweed	Prefers wet moist soils(wildflower.org). Stems arise from fleshy rhizomes, as vegetative reproduction is common [38].	Primary/dominant root is modified into tuber-like structures when colonized with mycorrhizae.
	*A*. *tuberosa*	Butterfly milkweed	Thick woody orange-brown tap-root that serves for C storage, attachment, and perennation. This species produces smaller lateral roots [40]. Many stems arise from a single root stock [38]	Primary/dominant root is modified into tuber-like structures when colonized with mycorrhizae.
	*A*. *viridis*	Spider milkweed	Prefers moist soils (wildflower.org). Root cardenolides in this species are four times higher than those of common milkweed [41].	Primary/dominant root is modified into tuber-like structures, but, in some cases, the tuber-like root was less elongated and more gall-like when colonized with mycorrhizae.

## Data Availability

Data shared upon request.

## Supplementary Materials

The following are available online at https://www.mdpi.com/article/10.3390/plants10112474/s1, Table S1. Code of Isolates. Table S2. Biomass explained by CO_2_, mycorrhizae, species, and growth chamber. Significant codes are for alpha thresholds. Table S3. Root morph. explained by CO_2_, mycorrhizae, species and growth chamber. Significant codes are for alpha thresholds. Figure S1 The effect of CO_2_ and mycorrhizae on fibrous root systems. Irrespective of CO_2_ regime, +mycorrhizae yielded a larger effect on tap-root systems. Table S4. GenBank Accession table.
