# Supplementary material for: Enriched CO2 and Root-Associated Fungi (Mycorrhizae) Yield Inverse Effects on Plant Mass and Root Morphology in Six Asclepias Species
Source: Plants (Basel). 2021 Nov 16;10(11):2474. doi: 10.3390/plants10112474 (PMC8617772; doi:10.3390/plants10112474)
Supplement: Supplementary file 1 [file plants-10-02474-s001.zip › plants-1404780-supplementary.pdf]

Supplementary Materials:

Table S1. Code of Isolates.

Mycorrhizal isolate	Isolation year	Isolate ID
Claroideoglomus claroideum	2014	87–5C
Claroideoglomus lamellosum	2017	57–4G
Glomus species	2017	69–6A
Racocetra fulgida	2017	57–4G

Table S2. Biomass explained by CO2, mycorrhizae, species, and growth chamber. Significant codes are for alpha thresholds.

S2: Analysis of Variance (ANOVA)

Source of biomass variation

	Df	Sum Sq	Mean Sq	F value	Pr(>F)	
CO2	1	52.08	52.08	8.5256	0.00407	**
Mycorrhizae	1	1436.55	1436.55	235.1612	< 2.2e-16	***
Species	5	547.96	109.59	17.9400	8.768e-14	***
Chamber	1	2.02	2.02	0.3302	0.56643	
CO2:Mycorrhizae	1	34.76	34.76	5.6905	0.01837	*
CO2:Species	5	77.62	15.52	2.5412	0.03091	*
Mycorrhizae:Species	5	483.63	96.73	15.8339	2.146e-12	***
CO2:Chamber	1	0.51	0.51	0.0834	0.77311	
Mycorrhizae:Chamber	1	3.14	3.14	0.5142	0.47450	
Species:Chamber	5	50.86	10.17	1.6652	0.14675	
CO2:Mycorrhizae:Species	5	60.78	12.16	1.9899	0.08361	.
CO2:Mycorrhizae:Chamber	1	0.01	0.01	0.0014	0.97003	
CO2:Species:Chamber	5	13.72	2.74	0.4492	0.81329	
Mycorrhizae:Species:Chamber	5	62.58	12.52	2.0487	0.07535	.
CO2:Mycorrhizae:Species:Chamber	5	11.95	2.39	0.3911	0.85427	
Residuals	143	873.56	6.11			

Signif. codes: 0 '***' 0.001 '**' 0.01 '*' 0.05 '.' 0.1 ' ' 1

Table S3. Root morph. explained by CO2, mycorrhizae, species and growth chamber. Significant codes are for alpha thresholds

S3: Analysis of Variance (ANOVA)

Source of relative transportive root variation

	Df	Sum Sq	Mean Sq	F value	Pr(>F)	
CO2	1	56	55.5	0.2289	0.63305	
Mycorrhizae	1	22277	22277.1	91.8808	< 2.2e-16	***
Species	5	10834	2166.9	8.9373	2.083e-07	***
Chamber	1	286	285.8	1.1786	0.27947	
CO2:Mycorrhizae	1	1111	1110.6	4.5807	0.03403	*
CO2:Species	5	1187	237.4	0.9791	0.43275	
Mycorrhizae:Species	5	11550	2309.9	9.5271	7.317e-08	***
CO2:Chamber	1	75	74.9	0.3090	0.57919	
Mycorrhizae:Chamber	1	638	638.3	2.6328	0.10688	
Species:Chamber	5	1275	255.0	1.0519	0.38978	
CO2:Mycorrhizae:Species	5	804	160.7	0.6629	0.65219	
CO2:Mycorrhizae:Chamber	1	108	108.5	0.4473	0.50469	
CO2:Species:Chamber	5	1613	322.6	1.3304	0.25465	
Mycorrhizae:Species:Chamber	5	304	60.8	0.2507	0.93889	
CO2:Mycorrhizae:Species:Chamber	5	639	127.8	0.5273	0.75531	
Residuals	143	34671	242.5			

Signif. codes: 0 '***' 0.001 '**' 0.01 '*' 0.05 '.' 0.1 ' ' 1

.

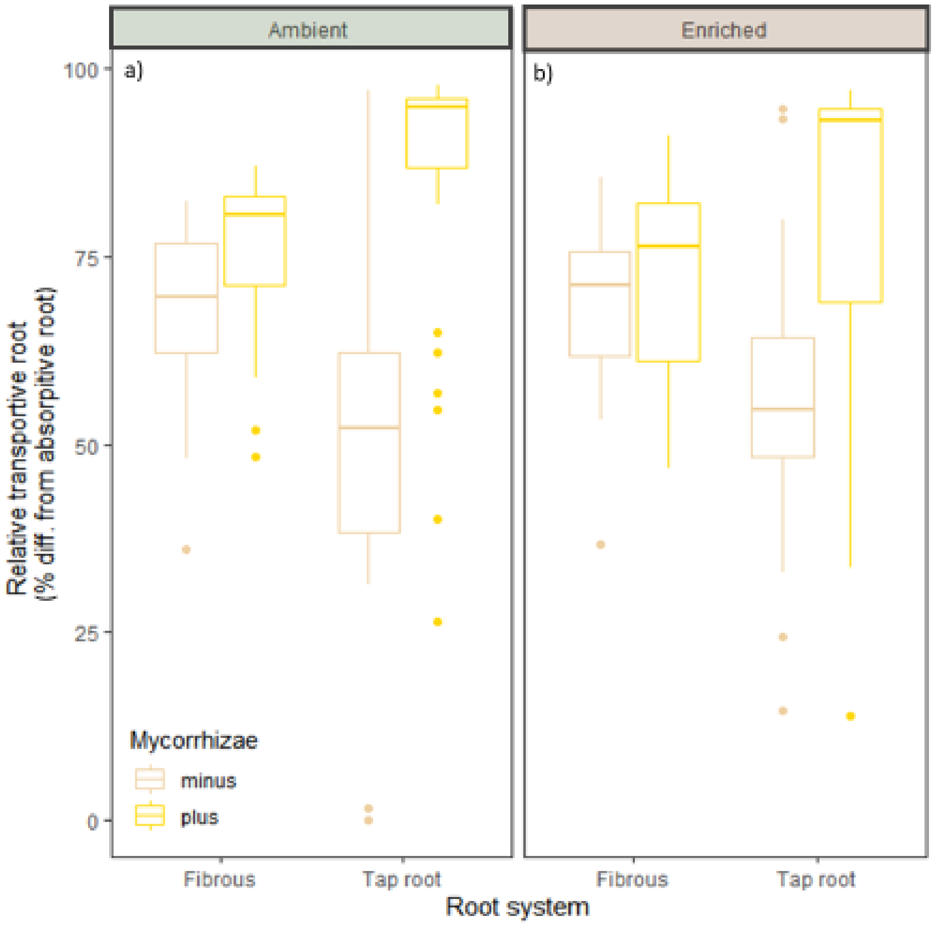

Figure S1. The effect of CO2 and mycorrhizae on fibrous root systems. Irrespective of CO2 regime, +mycorrhizae yielded a larger effect on tap-root systems.

Table S4. GenBank Accession table. Below Are the Corresponding Accessions Used to Construct the Phylogenetic Tree for the 6 Asclepias spp.

Asclepias rbcL **Accessions**	
A. incarnata	Accession: MK525246.1 [gene = rbcL] [protein = ribulose-1,5-bisphosphate carboxylase/oxygenase large subunit] [protein_id = QBP44096.1]
A. sullivantii	Accession: MK525250.1 [gene = rbcL] [protein = ribulose-1,5-bisphosphate carboxylase/oxygenase large subunit] [protein_id = QBP44100.1]
A. syriaca	Accession: HQ384906.1 [gene = rbcL] [protein = ribulose-1,5-bisphosphate carboxylase/oxygenase large subunit] [protein_id = AEH27042.1]
A. tuberosa	Accession: MN601492.1 [gene = rbcL] [protein = ribulose 1,5-bisphosphate carboxylase/oxygenase large subunit] [protein_id = QGV12460.1]
A. verticillata	Accession: KJ773290.1 [gene = rbcL] [protein = ribulose-1,5-bisphosphate carboxylase/oxygenase large subunit] [protein_id = AKG48691.1]
A. viridis	Accession: MH549741.1 [gene = rbcL] [protein = ribulose-1,5-bisphosphate carboxylase/oxygenase large subunit] [protein_id = QCW23980.1]
